# Determination and prediction of amino acid digestibility in rice bran for growing pigs

**DOI:** 10.5713/ab.25.0280

**Published:** 2025-08-25

**Authors:** Rui Li, Hui Tang, Menglong Deng, Xianji Jiang, Ganyi Feng, Xiaojie Liu, Ouyang Qin, Mingzhou Tian, Qiang Xiang

**Affiliations:** 1 Institute of Subtropical Agriculture, Chinese Academy of Sciences, Changsha, China; 2 College of Animal Science and Technology, Hunan Agricultural University, Hunan Co-Innovation Center of Animal Production Safety, Changsha, China

**Keywords:** Amino Acid, Growing Pigs, Prediction Model, Rice Bran, Standardized Ileal Digestibility

## Abstract

**Objective:**

This experiment was conducted to determine the apparent ileal digestibility or standardized ileal digestibility (SID) of crude protein (CP) and amino acids (AA) in 10 rice brans fed to pigs, and to construct predictive models for the SID of CP and AA based on the chemical composition of rice bran.

**Methods:**

Twenty-two healthy (Duroc×[Landrace×Yorkshire]) pigs equipped with ileal T-cannulas (initial body weight: 30±1.5 kg) were selected for this experiment. A replicated 11×3 incomplete Latin square design was adopted. The experiment consisted of 3 periods each lasting for 7 days and had 11 dietary treatment groups. The diets included 10 rice bran diets as well as a nitrogen-free diet for determining endogenous losses of AA, and each treatment group had 6 replicates. Titanium dioxide was added to each diet as an indicator at a concentration of 0.3%.

**Results:**

Except for dry matter (DM) and total phosphorus (TP), the coefficients of variation for the chemical components among 10 rice bran sources were all greater than 10%. The SID of CP, Lys, Met, Thr, and Trp in rice bran was 81.89%±6.23, 80.33%±2.21, 83.25%±5.51, 79.89%±5.68, and 72.12%±4.71, respectively. The best prediction equations for the SID of CP and four limiting AA in rice bran are as follows: SID_CP_ = 974.032–29.805TP–9.496DM (R^2^ = 0.88), SID_Lys_ = 471.278–9.267TP–4.245DM+1.401dummy variable (R^2^ = 0.92), SID_Met_ = 122.281–2.712CP (R^2^ = 0.51), SID_Thr_ = 51.864+2.204GE–1.324CF ([R^2^ = 0.97]; GE, gross energy; CF, crude fiber), and SID_Trp_ = 73.912–23.268Calcium–0.336NDF+0.318TS ([R^2^ = 0.96]; NDF, neutral detergent fiber; TS, total starch).

**Conclusion:**

There was significant variability among the chemical components of 10 different sources of rice bran. Moreover, GE, DM, CP, TP, TS, CF, and NDF could serve as crucial predictors for the SID of AA.

## INTRODUCTION

Feed cost accounts for more than 75% of pig production expenses, with energy ingredients such as corn constituting the largest proportion [1]. However, factors including African swine fever and geopolitical conflicts have led to a substantial surge in corn prices, driving continuous increases in feed costs and creating an urgent need for economically efficient alternative ingredients.

Rice bran, as a highly promising and cost-effective alternative energy ingredient for swine diets [2–4], boasts an annual global production of 83 million tons, 90% of which is utilized in livestock feed, demonstrating substantial resource advantages [4,5]. Rice bran is the primary byproduct generated during the milling (whitening) process for producing polished rice from brown rice after hulling, accounting for approximately 11% of the brown rice weight. Rice bran is composed of pericarp, tegmen, and aleurone of the grain [3]. As reported by the NRC [6], the digestible energy and metabolizable energy of rice bran on a fed basis are 12.97 MJ/kg and 12.54 MJ/kg, respectively. It can be used as an energy ingredient to replace corn. Additionally, rice bran is rich in essential fatty acids, minerals, vitamins, dietary fiber, antioxidants (e.g., tocopherols, tocotrienols, and γ-oryzanol), and high-quality protein [6–8]. The protein in rice bran, composed mainly of albumin (37.0%), globulin (36.0%), and glutelin (22.0%) [9], exhibits high digestibility, good biological value, and low allergenicity. Rice bran’s amino acid (AA) profile is well-balanced and contains notably higher levels of indispensable AA (particularly Lys) than other cereal brans [9]. Currently, significant variations exist in the chemical composition of rice bran compared to the ‘true value’ and ‘static parameter’ in existing databases due to factors such as rice bran variety, regional environment, and milling process. This poses challenges for achieving accurate nutritional formulation. Additionally, the limited studies on the AA digestibility of rice bran also affect the available use of rice bran in diets. Constructing dynamic prediction equations based on the chemical compositions of ingredients enables precise formulation design for animal feeds.

Therefore, the objective of this study was to determine the apparent ileal digestibility (AID) and standardized ileal digestibility (SID) of crude protein (CP) and AA in rice bran obtained from 10 different sources, while also developing a dynamic prediction equation for SID_AA_ in growing pigs based on the effective nutrient composition.

## MATERIALS AND METHODS

### Sources of rice bran samples

The study collected 10 samples of rice bran from six provinces in China, namely Jiangxi (with the sample number n = 2; Table 1), Heilongjiang (n = 1), Guangdong (n = 2), Shandong (n = 1), Hunan (n = 3), and Hebei (n = 1). The rice bran samples were crushed and sieved through 40-mesh screen. Then, they were stored at −20°C prior to chemical analysis.

### Animals, diets, and experimental design

Twenty-two healthy (Duroc×[Landrace×Yorkshire]) barrows with an initial body weight of 30±1.5 kg were selected for the experiment. Each pig was implanted with a T-shaped cannula at the terminal ileum following the method described by Stein et al [10]. A replicated 11×3 incomplete Latin square design was applied to pigs. The experiment comprised 3 consecutive periods. Each period lasted 7 days, including a 5-day adaptation period followed by a 2-day collection period for ileal digesta samples [1]. In the end, there were 6 replicates for each treatment group. Each pig was individually housed in a metabolic cage measuring 1.8 m×0.8 m×0.6 m and fed 10 diets containing rice bran as the sole nitrogen source, along with one nitrogen-free diet to determine basal endogenous losses of AA (Table 2). All the diets were supplemented with titanium dioxide (TiO_2_) at a concentration of 0.3% as an index and provided with vitamins and minerals according to the NRC [6] recommendations for growing pigs weighing between 25 to 50 kg. Throughout the experimental period, daily feed intake was calculated at 4% of each pig’s body weight and divided into two equal portions which were offered at 08:00 and 15:00, respectively, with free access to water provided at all times. At the end of each experimental period, all 22 pigs were reweighed to calculate their subsequent daily feed intake [10–12].

### Collection and processing of samples

As described by Stein et al [10,13], a pre-numbered self-sealing bag was secured over the open cannula with a rubber band when collecting samples. The bag was replaced every thirty minutes or when it reached one-third of its capacity. In addition, during the collection process, 5 mL of 10% (V/V) formic acid was added to each bag to prevent bacterial reproduction. After the collection was completed, these bags were immediately stored at −20°C. After one period ended, the samples were subjected to vacuum freeze drying for three days using the freeze-drying equipment (ACIENTZ-50F/A; Ningbo Xinzhi Freeze Drying Equipment). Finally, the freeze-dried digesta was crushed using an oscillating high-speed rotating mill (FW100; Tianjin Taisite). The crushing was performed with single 10-second operations, each followed by a cooling interval of over 120 seconds, repeated six times. The sample was then sieved through a 40-mesh sieve. Undersized digesta that failed to pass through the sieve was reprocessed using the same procedure, while the sieved sample was used for subsequent tests.

The contents of dry matter (DM, 930.15), ether extract (EE, 920.39), CP (984.13), crude ash (Ash, 942.05), calcium (Ca, 968.08), and total phosphorus (TP, 964.06) in 10 rice bran materials and 11 experimental diets were analyzed according to the AOAC [14] procedure. The contents of crude fiber (CF), neutral detergent fiber (NDF), and acid detergent fiber (ADF) were determined by automatic fiber analyzer (FT12; Gerhardt Analytical Instrument). The gross energy (GE) contents of samples were measured by an oxygen bomb automatic calorimeter (HXR-6000 Calorimeter; Hunan Huaxing Energy Sources Instrument). The content of total starch (TS) in rice bran and experimental diets was determined by K-TSTA kit (Megazyme). The contents of CP, DM, and AA in rice bran, experimental diets and prepared ileal digesta were determined. The contents of Met and Cys in samples were determined according to the AOAC [14] method [982.30 E(a)]. The content of Trp was determined by spectrophotometric method of GB/T 15400-2018 [15]. After pretreatment of 15 AA in samples with 6 *M* HCl, the content was determined by high-performance liquid chromatography (Agilent 1200; Agilent Technologies) [1,12,13].

### The mathematical formula for calculation

The index method was employed to determine the digestibility rate of AA in experimental diets, with the following calculation formula utilized [13]:


(1) $$\text{AID }(\%)=(1-{\left[{\text{AA}}_{\text{d}}\times \text{T}\right]}_{\text{r}}/[{\text{AA}}_{\text{r}}\times {\text{T}}_{\text{d}}])\times 100\%,$$

AA_d_ and T_d_ represent the concentrations of AA and TiO_2_ in the ileal digesta (g/kg of DM), respectively, while AA_r_ and T_r_ are the concentrations of AA and TiO_2_ in the rice bran diets (g/kg of DM), respectively. The same equation was used to calculate the AID of CP:


(2) $${\text{IAA}}_{\text{end}}=({\text{AA}}_{\text{d}}\times [{\text{T}}_{\text{r}}/{\text{T}}_{\text{d}}]),$$

where IAA_end_ represents the basal endogenous loss of each AA (g/kg of DM intake) and AA_d_ and T_d_ represent the concentrations of AA and TiO_2_ in the ileal digesta from the growing pigs fed the nitrogen-free diet, respectively. The T_r_ represents the concentration of TiO_2_ in the nitrogen-free diet. The same equation was used to calculate the endogenous loss of CP.


(3) $$\text{SID }(\%)=(\text{AID}+[{\text{IAA}}_{\text{end}}/{\text{AA}}_{\text{r}}]\times 100\%).$$

### Statistical analysis

The variance equivalence and normality of the data were assessed using descriptive statistical procedures in IBM SPSS 22.0 (IBM). Outlier identification and analysis were performed using Z-scores. The correlation between the chemical composition of rice bran samples and the SID values of Lys, Met, Thr, and Trp was analyzed using the CORR procedure in SPSS 22.0. Stepwise regression analysis in SPSS 22.0 was conducted to develop a predictive model for SID_AA_ (Lys, Met, Thr, and Trp) based on ten rice bran samples. The optimal model was selected based on relative standard deviation (RSD) values, maximum coefficient of determination (R^2^) value, as well as significant differences with p-values below 0.05 [1,12]. Additionally, during the development of the predictive model, a dummy variable (DV) was introduced into the set of independent variables to differentiate between full-fat rice bran and defatted rice bran. The DV assumes values of 0 and 1, corresponding to defatted rice bran (0) and full-fat rice bran (1), respectively, in this study. In our study, significant differences are indicated as p<0.05 and highly significant differences as p<0.01.

## RESULTS

### The chemical composition and amino acid profile of rice bran and its diets

On a fed basis, the content of CP, DM, GE, Ash, EE, Ca, TP, TS, CF, NDF, ADF, Lys, Met, Thr, and Trp in 10 rice bran averaged 14.51%±1.45, 88.32%±0.34, 16.52 MJ/kg±1.72, 9.19% ±1.65, 5.15%±5.65, 0.22%±0.22, 1.79%±0.16, 29.95%±4.49, 6.34%±1.93, 22.32%±4.43, 6.94%±2.87, 0.86%±0.10, 0.14%±0.02, 0.52%±0.06, and 0.19%±0.02, respectively (Table 3). The chemical components of the 10 rice bran samples exhibited great variability. Except for DM and TP, all coefficients of variation (CV) values for GE, CP, Ash, EE, Ca, TS, CF, NDF, ADF, Lys, Met, Thr, and Trp exceeded 10%. Notably, DM had the smallest CV value at 0.39%, while EE displayed the largest variability at 109.82%. On a fed basis, the content of Lys, Met, Thr, and Trp in 10 diets averaged 0.29%±0.03, 0.05%±0.01, 0.19%±0.02, and 0.05%±0.01, respectively (Table 4). Among these four limiting AA, only the CV for Met and Trp exceeded 10%. In addition, we conducted correlation analysis and established regression equations specifically for the 7 defatted rice bran samples, with details provided in Supplement 1 and Supplement 2.

### Apparent ileal digestibility and standardized ileal digestibility of crude protein and amino acid

The average values of AID_CP_, AID_Lys_, AID_Met_, AID_Thr_, and AID_Trp_ in 10 rice bran were 50.46%±6.35, 66.64%±2.22, 61.67%±5.19, 50.85%±4.70, and 58.79%±5.76 (Table 5), while the average values of SID_CP_, SID_Lys_, SID_Met_, SID_Thr_, and SID_Trp_ were 81.89%±6.23, 80.33%±2.21, 83.25%±5.51, 79.89%±5.68, and 72.12%±4.71 (Table 6), respectively. Except for Lys, the AID and SID of CP, Met, Thr, and Trp in the 10 rice bran samples all showed differences (p<0.01).

### Correlation analysis and prediction equations for standardized ileal digestibility of crude protein and amino acid

**Figure 1:**
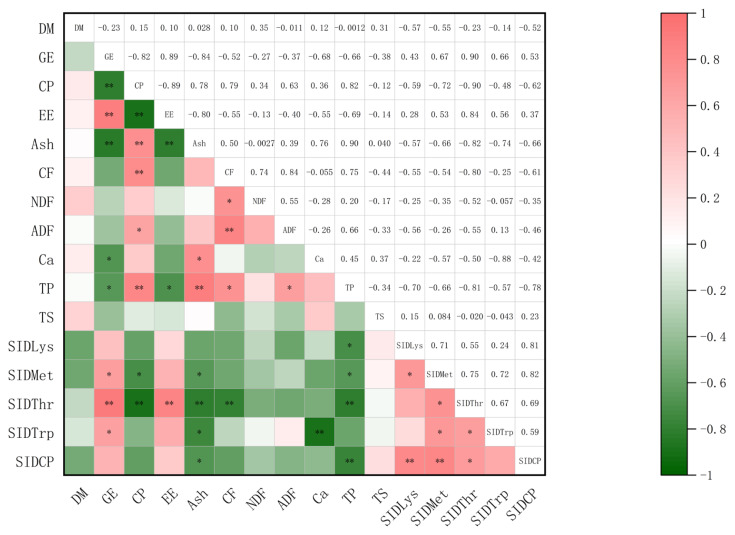
The correlation coefficients between the chemical components of 10 rice bran samples and the standardized ileal digestibility (SID) of crude protein (CP) and the first four limiting amino acids (AA). * p≤0.05, ** p≤0.01. DM, dry matter; GE, gross energy; EE, ether extract; Ash, crude ash; CF, crude fiber; NDF, neutral detergent fiber; ADF, acid detergent fiber; Ca, Calcium; TP, total phosphorus; TS, total starch; SID_CP_, SID_Lys_, SID_Met_, SID_Thr_, and SID_Trp_, SID of CP, Lys, Met, Thr, and Trp, respectively.

As shown in Figure 1, a correlation analysis was conducted on the chemical components of rice bran, the SID_CP_ and the SID of four limiting AA. The results showed that the SID_CP_ was negatively correlated with the contents of TP (p<0.01) and Ash (p<0.05). The SID_Lys_ was positively correlated with SID_CP_ (p<0.01) and negatively correlated with the content of TP (p<0.05). The SID_Met_ showed a positive correlation with GE (p<0.05), SID_CP_ (p<0.01), and SID_Lys_ (p<0.05), and a negative correlation with the contents of CP (p<0.05), Ash (p<0.05) and TP (p<0.05). The SID_Thr_ was positively correlated with GE (p<0.01), the content of EE (p<0.01), SID_CP_ (p<0.05), and SID_Met_ (p<0.05), and negatively correlated with the contents of CP (p<0.01), Ash (p<0.01), CF (p<0.01), and TP (p<0.01). The SID_Trp_ showed a positive correlation with GE (p<0.05), SID_Met_ (p<0.05) and SID_Thr_ (p<0.05), and a negative correlation with the contents of Ash (p<0.05) and Ca (p<0.01).

The prediction equations for estimating the SID of CP, Lys, Met, Thr, and Trp in rice bran were developed based on the levels of effective chemical components. The optimized prediction equations obtained were as follows: SID_CP_ = 974.032–29.805TP–9.946DM (RSD = 2.44, R^2^ = 0.88, and p<0.001; Table 7), SID_Lys_ = 471.278–9.267TP–4.245DM+1.401DV (RSD = 0.77, R^2^ = 0.92, and p = 0.001), SID_Met_ = 122.281–2.712CP (RSD = 4.08, R^2^ = 0.51, and p = 0.020), SID_Thr_ = 51.864+ 2.204GE–1.324CF (RSD = 1.21, R^2^ = 0.97, and p<0.001), and SID_Trp_ = 73.912–23.268Ca–0.336NDF+0.318TS (RSD = 1.10, R^2^ = 0.96, and p = 0.011).

## DISCUSSION

### Chemical composition and amino acid profile of rice bran

The chemical components of the rice bran samples were within the range of the published table values [6,16–18]. In this study, the CV of DM and TP were lower than 10%, while those of CP and GE were close to 10%, indicating good homogeneity of these chemical components. The CP content or GE of some samples is relatively high, but still lower than the reported values of Huang et al [17] and Casas and Stein [19]. The CV of the rest of the chemical components in our samples obviously exceeded 10%. Among them, the CV of CF, ADF, Ca, and EE all exceeded 30%. Chemical composition and AA content of rice bran will vary greatly due to processing conditions, climatic conditions and production regions. During the rice milling process, different milling degrees will also cause changes in the chemical components and AA contents of rice bran. It has been reported [20] that the rice bran produced with an 8% milling degree has the lowest contents of protein, fat, and CF. The rice bran produced with a 6% milling degree has the highest mineral content, while the rice bran produced with a 2% milling degree has the highest vitamin content [20]. Moreover, the bran produced by friction mills can remove the aleurone layer more effectively and has a relatively high fat content, and abrasive mills remove the endosperm with a higher starch content at the same degree of milling [20]. The defatting of rice bran directly affects the changes in the content of chemical components. Since both full-fat rice bran and defatted rice bran were present in our experiment simultaneously, the CV of EE was as high as 109.82%. As can be seen from Table 3, for the samples with high EE content, their GE was mostly on the high side.

In this study, the average content range of 18 AA in 10 rice bran was between 0.14% and 1.89%, basically within the reported range of the tabulated values [6,16–18]. Among them, the average content of Lys (0.86%) was higher than the reported values of the NRC [6] (0.67%) and the Nutrient Requirements of Swine in China [16] (0.60%), but was basically within the range reported by previous researchers [18,21]. The average content of Thr (0.52%) was basically consistent with the reported values of NRC [6] (0.56%) and the Nutrient Requirements of Swine in China [16] (0.46%). The average content of Met (0.14%) was lower than the reported values in the NRC [6] (0.3%) and Chinese Swine Nutrient Requirements [16] (0.24%), but within the range of values reported in the literature [21–23]. The average content of Trp (0.19%) fell within the range of values reported by the NRC [6] (0.19%) and Nutrient Requirements of Swine in China [16] (0.14%). In our study, the CV of His, Ile, Cys, and Tyr in the 10 rice bran was within 10%, the CV of the other AA was basically greater than 10%. This indicates that the contents of AA in different rice bran fluctuated greatly and were unstable. From the perspective of plant morphology, rice bran in the strict sense is composed of the aleurone layer, nucellus layer, seed coat, pericarp layer, and several sub-layers among them [20]. However, in the actual production process of rice bran samples, rice bran usually contains rice germ and broken rice, which will directly affect the chemical components and AA contents of rice bran [20].

Accurately estimating the utilization rate of AA in diets is the basis for implementing the low-protein diet system. However, due to the large quantity of rice bran produced around the world, different processing techniques, and complex sources, it is difficult to identify and investigate. Therefore, it is necessary to measure its chemical components and AA contents, and to construct a dynamic prediction equation for the SID_AA_ of rice bran for pigs based on its chemical component contents [1,24]. In this study, multiple routine chemical component indicators and AA contents among different samples had relatively high fluctuation levels, which ensured the feasibility of establishing correlations and prediction equations and was conducive to the progress of subsequent experiments.

### Standardized ileal digestibility of amino acid in rice bran

The SID is obtained by correcting the AID for basic endogenous losses [11,13]. This endows the SID values with the property of additivity and makes it the most recognized and effective method for estimating the availability of AA in the field of animal nutrition [24,25]. In our study, the average AID and SID values of AA and CP in the 10 rice bran samples were basically within the range of the published tabulated values [6,16,18,23,26]. Park et al [27] analyzed and summarized the basal endogenous losses of CP and AA in pigs fed nitrogen-free diets, and the basal endogenous losses of CP and AA in this study fall within the range of values presented in their summary table.

In the study by Casas et al [28], rice bran showed reduced SID_AA_ compared to brown rice. Kinh et al [29] further reported a hierarchy of SID_AA_ values in rice derivatives: broken rice>rice bran>paddy rice, attributing this pattern to variations in fiber content among these byproducts.

In contrast, EE content in rice bran positively modulates AA utilization. Casas et al [30] observed higher AID_AA_ in full-fat rice bran than in defatted samples. In Ouyang et al’s prediction equation [13], the EE content in brown rice was positively correlated with its AA digestibility. Cervantes-Pahm et al [31] showed that adding oil can increase the SID_AA_ in soybean meal. These studies are consistent with the phenomenon we observed. Cervantes-Pahm et al [31] hypothesized that this phenomenon may stem from two aspects: first, EE slows down the gastric emptying rate, thereby prolonging the contact time between feed protein and proteases; second, the presence of EE in the small intestine may reduce the passage rate of ingested feed, thus providing more sufficient time for the absorption of AA and peptides.

In our study, correlation analyses between EE content and the SID of CP or four limiting AA revealed a positive association between EE content and SID_Thr_ (p<0.01). Furthermore, SID_AA_ of rice bran exhibited significant negative or positive correlations with multiple chemical constituents, including DM, TP, EE, NDF, and ADF. These results indicate that AA digestibility in rice bran is influenced by interactions among multiple chemical components, enabling dynamic prediction of SID_AA_ based on compositional parameters.

### Correlation analysis and prediction equations for standardized ileal digestibility of amino acid in rice bran

Currently, the chemical composition of ingredients has been confirmed by multiple studies to be related to their digestibility [1,12,32]. Prediction equations for digestible energy, metabolizable energy, and AA digestibility in pig feeds have been widely developed. By referring to previous studies [1,12,32], we established prediction equations for the SID_AA_ of rice bran based on its chemical components using Pearson correlation analysis and stepwise linear regression methods.

Our study found that the SID values of CP and Lys were negatively correlated with the TP content, and both TP and DM were the key predictors for them at the same time. The negative impact of the TP content on the SID values of CP and Lys should be associated with phytic acid. Phytic acid is the main anti-nutritional factor in rice bran [33]. When the TP is relatively high, the content of phytic acid tends to be relatively high as well. Phytic acid has multiple phosphate groups [33]. It has been reported that it can combine with mineral ions and free AA to form highly stable and insoluble phytic acid-mineral-AA complexes [33,34]. Moreover, considering the exposed positively charged ɛ-amino group of Lys [24], it is more likely to combine with phytic acid to form complexes. As noted above, the positively charged ɛ-amino group of Lys endows it with strong reducing ability, rendering its digestibility highly sensitive to processing conditions. DV, serving as a categorical indicator that distinguishes full-fat from defatted rice bran, precisely captures this processing disparity—explaining its emergence as a predictor in the SID_Lys_ equation.

Li et al [35] found in paddy rice that DM content made a negative contribution to the prediction equations of SID_CP_ and SID_Lys_. Higher DM content can lead to a relatively lower water content in food and a reduction in the number of water molecules around starch molecules. At this time, the opportunities for hydrogen bonds to form between the hydroxyl groups on starch molecules will increase [36], resulting in a denser food structure. Digestive enzymes can function better in a solution because enzymes need to diffuse through water to access substrates. Higher DM content makes it more difficult for digestive fluids to penetrate, thus impeding the contact between enzymes and substrates. Moreover, it brings phytic acid, minerals, and AA closer together, making it easier for them to form indigestible complexes. These factors may be the reasons why DM becomes a key predictor in the prediction equations of SID_CP_ and SID_Lys_. However, in our study, the CV for DM in rice bran was only 0.39%. When developing predictive models, the inclusion of an independent variable with such low variability compromises the stability of its practical application. For instance, if predicted rice bran DM values fall outside the parameter range established during modeling, the results may severely deviate from actual digestibility. Furthermore, DM itself may merely indirectly represent other unmeasured variables that could have more direct impacts on outcomes.

The key predictor of the SID_Met_ prediction equation is the CP content, and it is negatively correlated with SID_Met_. In the Nutrient Requirements of Swine in China [16], CP content is usually regarded as a prediction factor with a positive contribution. Li et al [1] also found that the SID_Met_ in barley was positively correlated with the CP content. However, considering that in our study, the CP content showed a very strong positive correlation with both the fiber content and the TP content simultaneously, this would lead to the CP content being able to replace the latter two, and thus exhibited a stronger fitting with SID_Met_ in the linear fitting.

The fiber component serves as a key predictor for both the SID_Thr_ and SID_Trp_, playing a negative contributory role. In previous reports, the fiber fraction was found to be negatively correlated with the ileal digestibility of AA, which is consistent with the results of our study [24,37]. There are multiple mechanisms by which the fiber fraction reduces the digestibility of AA. These include increasing the viscosity of digesta to slow down mass transfer and enzymatic reactions, augmenting endogenous AA losses, and combining with specific AA to form complexes that are difficult to hydrolyze, all of which act to reduce the digestibility of CP and AA [13].

In this study, we obtained two prediction equations for SID_CP_, two equations for SID_Lys_, one equation for SID_Met_, two equations for SID_Thr_, and three equations for SID_Trp_ from 10 rice bran samples respectively. Our results indicate that phytic acid, moisture content, and fiber fraction may be important factors affecting the digestibility of AA in rice bran.

### Validate the prediction equations for standardized ileal digestibility_crude protein_ and standardized ileal digestibility_amino acid_ of rice bran based on database

Due to the absence of some parameters in the NRC [6], we used the prediction equations and the parameters of full-fat rice bran in the Nutrient Requirements of Swine in China [16] to predict SID_CP_, SID_Lys_, SID_Met_, SID_Thr_, and SID_Trp_. The predicted values were 70.72%, 75.45%, 82.41%, 82.77%, and 71.91%, respectively, while the reported values in the Nutrient Requirements of Swine in China [16] were 75%, 75%, 80%, 80%, and 71% respectively. The predicted values are close to the reported values, but this validation is relatively basic. Future studies should conduct more rigorous validation of the equation through animal experiments. Additionally, Choi et al [38] used regression analysis to validate the digestible energy prediction equation, which is also a scientific validation method: they compared the known digestible energy values from the literature with those predicted by the equation, and judged the accuracy of the equation by checking whether the intercept was not significantly different from 0 and the slope was not significantly different from 1. However, when we attempted to use this method to validate the prediction equations for AA digestibility in rice bran, we faced the problem of insufficient data. This indicates that research on AA digestibility in rice bran still needs to be further advanced.

## CONCLUSION

In summary, the chemical composition of 10 rice bran samples exhibited significant differences. The SID of CP and the first four limiting AA could be estimated from the analyzed contents of GE, TP, DM, CP, CF, NDF, and TS.

## Figures and Tables

**Table 1:** Sources of rice bran

Sample number	Type	Sources
RB1	Defatted rice bran	Nanchang city, Jiangxi
RB2	Defatted rice bran	Harbin city, Heilongjiang
RB3	Defatted rice bran	Foshan city, Guangdong
RB4	Full-fat rice bran	Zhaoqing city, Guangdong
RB5	Full-fat rice bran	Yichun city, Jiangxi
RB6	Defatted rice bran	Rizhao city, Shandong
RB7	Defatted rice bran	Zhuzhou city, Hunan
RB8	Defatted rice bran	Changsha city, Hunan
RB9	Full-fat rice bran	Changsha city, Hunan
RB10	Defatted rice bran	Xiangyang city, Hubei

**Table 2:** Composition of rice bran experimental diet (as-fed basis, %)

Items	Nitrogen-free diet	Experimental diet
Corn starch	78.90	42.90
Rice bran	0.00	40.00
Sucrose	10.00	10.00
Soybean oil	3.00	3.00
Cellulose acetate	4.00	0.00
Limestone	0.50	0.50
CaH_2_PO_4_	1.90	1.90
TiO^2^	0.30	0.30
NaCl	0.40	0.40
Vitamin and mineral premix^1)^	0.50	0.50
K_2_CO_3_	0.40	0.40
MgO	0.10	0.10
Total	100.00	100.00

1) Per kilogram of the diet, the following vitamins and minerals are provided: vit A 4,200 IU, vit B_1_ 3.4 mg, vit B_2_ 5.63 mg, vit B_3_ 28 mg, vit B_12_ 23 μg, vit D_3_ 400 IU, vit E 36 IU, vit K_3_ 1.2 mg, choline chloride 1.00 g, folic acid 0.8 mg, vit B_5_ 20.5 mg, vit B_6_ 2.7 mg, vit H 0.18 mg, Cu (as copper sulfate) 70 mg, Mn (as manganese sulfate) 40.0 mg, Fe (as ferrous sulfate) 70.0 mg, Zn (as zinc sulfate) 70.0 mg, Se (as sodium selenite) 0.3 mg, I (as potassium iodide) 0.3 mg.

**Table 3:** Analyzed chemical composition of 10 rice bran samples (as-fed basis, %)

Items	Rice bran number										Min	Max	Mean	CV (%)

	RB1	RB2	RB3	RB4	RB5	RB6	RB7	RB8	RB9	RB10				
DM	88.34	88.61	88.27	88.3	88.56	88.39	88.33	88.45	88.56	87.4	87.40	88.61	88.32	0.39
GE (MJ/kg)	15.51	15.31	14.69	18.22	18.98	15.53	15.28	14.91	18.4	18.35	14.69	18.98	16.52	10.44
CP	16.29	15.13	14.99	11.84	13.83	16.02	15.33	15.35	12.71	13.63	11.84	16.29	14.51	10.02
EE	1.35	0.75	1.13	14.37	12.20	2.29	1.31	0.55	12.82	4.72	0.55	14.37	5.15	109.82
Ash	10.31	9.01	11.49	7.71	8.24	10.18	9.11	11.32	6.29	8.28	6.29	11.49	9.19	17.92
CF	9.97	5.93	5.64	4.23	5.00	9.67	6.06	6.29	5.24	5.32	4.23	9.97	6.34	30.46
NDF	29.35	22.35	18.74	21.58	16.72	28.24	21.72	20.43	26.68	17.34	16.72	29.35	22.32	19.87
ADF	11.97	7.06	6.66	6.06	6.18	11.59	7.08	3.67	3.19	5.94	3.19	11.97	6.94	41.36
Ca	0.13	0.22	0.64	0.08	0.07	0.11	0.20	0.61	0.08	0.08	0.07	0.64	0.22	98.50
TP	2.01	1.74	1.94	1.62	1.77	1.98	1.70	1.91	1.52	1.73	1.52	2.01	1.79	9.07
TS	25.01	36.14	32.48	34.34	25.04	25.9	35.22	31.18	28.97	25.26	25.01	36.14	29.95	14.98
Indispensable amino acids (%)														
Arginine	1.01	1.01	1.11	0.85	0.93	1.10	0.98	1.11	0.8	0.94	0.8	1.11	0.98	10.88
Histidine	0.65	0.56	0.62	0.53	0.55	0.66	0.55	0.61	0.51	0.57	0.51	0.66	0.58	8.79
Isoleucine	0.56	0.57	0.57	0.46	0.49	0.59	0.52	0.57	0.44	0.54	0.44	0.59	0.53	9.74
Leucine	1.05	1.06	1.07	0.84	0.90	1.13	0.98	1.09	0.81	0.95	0.81	1.13	0.99	11.15
Lysine	0.93	0.88	0.95	0.70	0.80	1.02	0.85	0.93	0.71	0.82	0.70	1.02	0.86	12.1
Methionine	0.11	0.13	0.15	0.14	0.13	0.11	0.16	0.15	0.16	0.15	0.11	0.16	0.14	13.33
Phenylalanine	0.70	0.70	0.7	0.57	0.60	0.75	0.65	0.71	0.54	0.65	0.54	0.75	0.66	10.35
Threonine	0.56	0.56	0.57	0.44	0.48	0.60	0.51	0.58	0.43	0.51	0.43	0.60	0.52	11.35
Tryptophan	0.19	0.22	0.19	0.17	0.19	0.18	0.19	0.15	0.18	0.23	0.15	0.23	0.19	12.22
Valine	0.82	0.83	0.83	0.65	0.71	0.86	0.76	0.84	0.62	0.74	0.62	0.86	0.77	11.01
Dispensable amino acids (%)														
Alanine	0.89	0.89	0.91	0.68	0.73	0.96	0.82	0.91	0.66	0.76	0.66	0.96	0.82	13.00
Aspartate	1.49	1.43	1.47	1.07	1.18	1.60	1.32	1.47	1.01	1.14	1.01	1.60	1.32	15.49
Cystine	0.20	0.21	0.21	0.22	0.22	0.21	0.21	0.22	0.20	0.23	0.2	0.23	0.21	4.45
Glutamine	1.97	2.16	2.17	1.56	1.63	2.13	1.98	2.21	1.49	1.59	1.49	2.21	1.89	15.31
Glycine	0.76	0.77	0.79	0.58	0.64	0.82	0.7	0.79	0.56	0.66	0.56	0.82	0.71	13.15
Proline	0.80	0.65	0.79	0.57	0.61	0.81	0.67	0.72	0.59	0.63	0.57	0.81	0.68	13.21
Serine	0.60	0.61	0.61	0.47	0.52	0.67	0.56	0.62	0.46	0.56	0.46	0.67	0.57	11.93
Tyrosine	0.35	0.37	0.41	0.36	0.39	0.37	0.37	0.42	0.33	0.42	0.33	0.42	0.38	8.01

CV, coefficient of variation; DM, dry matter; GE, gross energy; CP, crude protein; EE, ether extract; Ash, crude ash; CF, crude fiber; NDF, neutral detergent fiber; ADF, acid detergent fiber; Ca, Calcium; TP, total phosphorus; TS, total starch.

**Table 4:** Analyzed chemical composition of experiment diets (as-fed basis, %)

Items	Rice bran number										Min	Max	Mean	CV (%)

	RB1	RB2	RB3	RB4	RB5	RB6	RB7	RB8	RB9	RB10				
DM	90.02	90.31	90.25	90.04	90.18	90.33	90.06	90.15	90.34	90.28	90.02	90.34	90.20	0.14
GE (MJ/kg)	15.45	15.39	15.17	16.55	16.65	15.72	15.5	15.12	16.8	16.82	15.12	16.82	15.92	4.41
CP	6.53	5.43	4.89	5.19	5.18	6.36	6.35	5.97	4.36	3.27	3.27	6.53	5.35	19.07
EE	3.57	2.09	1.36	7.11	5.89	2.56	2.08	2.47	5.27	6.24	1.36	7.11	3.86	53.59
Ash	8.10	7.34	7.92	6.50	6.57	7.23	6.51	7.45	5.59	6.58	5.59	8.10	6.98	10.96
CF	4.24	2.68	2.33	2.33	2.08	3.48	2.3	2.42	2.10	2.02	2.02	4.24	2.60	27.50
NDF	11.8	10.16	7.36	8.56	7.77	12.34	8.9	8.99	10.3	7.76	7.36	12.34	9.39	18.24
ADF	4.40	3.22	2.53	2.29	2.77	4.97	2.66	3.04	2.65	2.33	2.29	4.97	3.09	29.15
TS	44.48	51.93	51.59	51.61	51.99	48.17	53.49	52.44	43.03	42.55	42.55	53.49	49.13	8.62
Indispensable amino acids (%)														
Arginine	0.38	0.33	0.34	0.35	0.33	0.37	0.36	0.38	0.36	0.4	0.33	0.4	0.36	6.40
Histidine	0.22	0.20	0.20	0.22	0.21	0.26	0.24	0.23	0.19	0.22	0.19	0.26	0.22	9.17
Isoleucine	0.20	0.18	0.18	0.18	0.19	0.21	0.21	0.20	0.18	0.19	0.18	0.21	0.19	6.41
Leucine	0.40	0.35	0.34	0.34	0.34	0.40	0.39	0.39	0.35	0.37	0.34	0.40	0.37	7.06
Lysine	0.29	0.27	0.27	0.27	0.26	0.34	0.31	0.32	0.28	0.25	0.25	0.34	0.29	9.75
Methionine	0.04	0.04	0.04	0.06	0.06	0.05	0.07	0.07	0.06	0.05	0.04	0.07	0.05	19.28
Phenylalanine	0.27	0.23	0.22	0.22	0.23	0.26	0.26	0.26	0.23	0.24	0.22	0.27	0.24	6.99
Threonine	0.22	0.19	0.18	0.18	0.17	0.21	0.20	0.20	0.19	0.20	0.17	0.22	0.19	7.24
Tryptophan	0.08	0.10	0.07	0.07	0.09	0.09	0.09	0.07	0.07	0.09	0.07	0.10	0.05	12.34
Valine	0.30	0.27	0.26	0.26	0.26	0.31	0.29	0.30	0.27	0.28	0.26	0.31	0.28	6.79
Dispensable amino acids (%)														
Alanine	0.34	0.30	0.29	0.28	0.28	0.34	0.32	0.33	0.29	0.31	0.28	0.34	0.31	7.85
Aspartate	0.58	0.48	0.47	0.44	0.47	0.58	0.52	0.53	0.46	0.51	0.44	0.58	0.50	9.55
Cystine	0.06	0.05	0.05	0.05	0.05	0.05	0.05	0.05	0.06	0.07	0.05	0.07	0.06	10.43
Glutamine	0.82	0.74	0.70	0.68	0.69	0.78	0.80	0.80	0.70	0.76	0.68	0.82	0.75	6.93
Glycine	0.29	0.26	0.25	0.24	0.24	0.29	0.27	0.29	0.25	0.27	0.24	0.29	0.26	7.84
Proline	0.24	0.21	0.21	0.23	0.20	0.28	0.26	0.26	0.20	0.21	0.20	0.28	0.23	11.73
Serine	0.26	0.22	0.20	0.19	0.20	0.22	0.21	0.22	0.21	0.24	0.19	0.26	0.22	9.69
Tyrosine	0.11	0.11	0.12	0.13	0.13	0.12	0.11	0.13	0.13	0.15	0.11	0.15	0.12	9.75

CV, coefficient of variation; DM, dry matter; GE, gross energy; CP, crude protein; EE, ether extract; Ash, crude ash; CF, crude fiber; NDF, neutral detergent fiber; ADF, acid detergent fiber; TS, total starch.

**Table 5:** Apparent ileal digestibility of crude protein (CP) and amino acids in rice bran fed to growing pigs (%)

Items	Rice bran number										Mean	SEM	p-value

	RB1	RB2	RB3	RB4	RB5	RB6	RB7	RB8	RB9	RB10			
CP (%)	51.93^bc^	51.20^bc^	42.64^d^	56.61^b^	48.41^cd^	48.44^cd^	63.92^a^	51.07^bc^	47.97^cd^	42.39^d^	50.46	1.07	<0.001
Indispensable amino acids (%)													
Arginine	68.02^c^	69.13^bc^	68.11^c^	73.40^abc^	69.51^bc^	67.55^c^	74.97^abc^	73.53^abc^	76.63^a^	77.39^a^	71.83	0.85	0.023
Histidine	87.74	84.09	83.49	86.11	86.56	87.42	86.32	82.92	82.52	83.21	85.04	0.56	0.245
Isoleucine	63.91^c^	63.76^c^	64.67^c^	64.68^c^	66.83^bc^	63.07^a^	73.72^c^	71.55^ab^	75.18^a^	77.00^a^	68.44	0.85	<0.001
Leucine	58.58^d^	57.03^d^	55.47^d^	65.35^c^	65.86^c^	57.77^d^	73.81^a^	68.22^bc^	71.24^ab^	73.61^a^	64.69	1.01	<0.001
Lysine	63.54	65.49	65.20	66.80	63.10	67.75	68.81	67.49	69.29	68.92	66.64	0.60	0.186
Methionine	57.77^b^	58.35^b^	55.96^b^	73.17^a^	62.55^ab^	57.38^b^	65.67^ab^	59.26^b^	61.69^ab^	64.89^ab^	61.67	1.25	0.059
Phenylalanine	62.89^bc^	62.89^bc^	58.72^c^	66.15^abc^	72.52^a^	59.87^c^	72.32^a^	67.13^abc^	70.36^ab^	70.43^ab^	66.66	0.99	0.002
Threonine	47.03^c^	48.01^c^	45.51^c^	55.99^ab^	55.06^ab^	46.56^c^	51.00^bc^	46.49^c^	55.02^ab^	57.82^a^	50.85	0.85	<0.001
Tryptophan	58.73^bc^	62.47^abc^	48.76^d^	62.57^abc^	64.35^a^	59.95^abc^	62.89^ab^	54.84^d^	60.87^c^	63.64^abc^	58.79	0.85	<0.001
Valine	56.01^ef^	58.14^e^	51.85^f^	64.25^cd^	68.64^bc^	60.51^de^	74.59^a^	67.53^bc^	66.61^bc^	70.23^ab^	63.84	0.99	<0.001
Dispensable amino acids (%)													
Alanine	53.91^c^	51.63^c^	50.92^c^	58.51^abc^	54.53^c^	56.38^bc^	64.86^a^	62.71^ab^	64.46^a^	66.47^a^	58.44	1.04	<0.001
Aspartate	47.32^de^	46.09^de^	43.27^e^	51.92^cd^	57.10^bc^	47.96^de^	67.83^a^	57.32^bc^	57.97^bc^	62.93^abc^	53.97	1.15	<0.001
Cystine	72.69^a^	56.69^b^	50.64^b^	72.54^a^	70.83^a^	66.24^a^	68.22^a^	56.83^b^	66.27^a^	68.62^a^	64.96	1.17	<0.001
Glutamine	58.46^ef^	61.14^de^	54.69^f^	67.35^bc^	68.89^abc^	60.84^de^	73.94^a^	66.64^cd^	73.21^ab^	73.46^ab^	65.86	1.03	<0.001
Glycine	38.08^cd^	36.49^d^	41.35^bcd^	40.22^bcd^	38.41^cd^	36.22^d^	50.11^a^	40.03^bcd^	43.82^abc^	46.46^ab^	41.12	0.84	<0.001
Proline	53.43^d^	63.90^ab^	64.45^ab^	68.52^a^	50.03^d^	60.35^bc^	63.82^ab^	51.99^d^	54.86^cd^	63.04^ab^	59.44	1.01	<0.001
Serine	52.03^bcd^	45.29^d^	49.81^cd^	47.19^d^	54.06^bcd^	46.83^d^	64.75^a^	57.68^abc^	60.01^ab^	64.75^a^	54.24	1.27	<0.001
Tyrosine	73.47^ab^	78.02^b^	60.76^ab^	77.64^ab^	80.10^a^	74.87^b^	78.40^a^	66.35^b^	71.32^ab^	77.39^a^	73.83	0.76	<0.001

a–f Means in the same row with no letter the same letter are not different at p<0.05.

SEM, standard error of the mean.

**Table 6:** Standardlized ileal digestibility of crude protein (CP) and amino acids in rice bran fed to growing pigs (%)

Items	Rice bran number										Mean	SEM	p-value

	RB1	RB2	RB3	RB4	RB5	RB6	RB7	RB8	RB9	RB10			
CP (%)	76.63^d^	80.97^bcd^	75.71^d^	87.67^ab^	79.56^cd^	73.89^d^	89.31^a^	78.13^cd^	85.10^abc^	91.90^a^	81.89	1.08	<0.001
Indispensable amino acids (%)													
Arginine	83.98	87.73	86.16	90.94	87.80	84.15	91.87	89.60	93.71	92.70	88.86	0.83	0.055
Histidine	97.34	94.78	94.29	95.74	96.81	95.84	95.14	92.35	93.83	93.02	94.91	0.54	0.570
Isoleucine	76.93^d^	78.19^d^	79.54^cd^	79.31^cd^	80.73^cd^	75.65^d^	86.36^ab^	84.59^bc^	89.63^ab^	90.83^a^	82.18	0.84	<0.001
Leucine	71.34^c^	71.67^c^	70.65^c^	80.47^b^	81.11^b^	70.56^c^	87.06^a^	81.43^b^	85.86^ab^	87.63^a^	78.78	1.01	<0.001
Lysine	76.97	79.85	79.55	81.20	78.05	79.31	81.35	79.60	83.05	84.33	80.33	0.60	0.190
Methionine	76.24^c^	82.35^bc^	77.12^c^	92.00^a^	82.80^bc^	81.76^bc^	84.35^b^	77.89^bc^	82.70^bc^	92.07^a^	83.25	0.94	<0.001
Phenylalanine	78.18^cd^	80.48^bcd^	77.11^cd^	84.37^abc^	90.54^a^	75.45^d^	88.32^ab^	83.20^abcd^	88.22^ab^	87.31^ab^	83.63	1.01	<0.001
Threonine	72.71^d^	77.88^cd^	77.05^cd^	86.65^a^	87.21^a^	73.65^cd^	79.52^bc^	74.07^cd^	84.82^ab^	85.34^ab^	79.89	0.93	<0.001
Tryptophan	71.53^b^	73.73^ab^	67.25^c^	77.50^a^	76.53^ab^	71.43^b^	75.15^ab^	62.97^c^	72.13^b^	74.94^ab^	72.12	0.74	<0.001
Valine	67.09^cd^	70.59^c^	64.62^d^	77.08^b^	81.42^ab^	71.30^c^	85.92^a^	78.75^b^	79.17^b^	82.04^ab^	75.80	0.99	<0.001
Dispensable amino acids (%)													
Alanine	67.51^c^	67.25^c^	66.99^c^	75.10^abc^	71.13^bc^	70.05^bc^	79.26^a^	76.78^ab^	80.56^a^	81.49^a^	73.61	1.03	<0.01
Aspartate	72.40^d^	76.28^d^	74.32^d^	84.93^c^	88.23^bc^	73.42^d^	95.99^a^	84.89^c^	90.11^abc^	91.55^ab^	83.21	1.21	<0.001
Cystine	95.56^a^	81.37^cd^	76.71^d^	98.30^a^	96.46^a^	91.26^ab^	93.33^ab^	82.91^cd^	87.48^bc^	88.18^bc^	89.15	1.12	<0.001
Glutamine	71.81^d^	75.98^cd^	70.29^d^	83.41^ab^	84.83^ab^	74.88^cd^	87.66^a^	80.31^bc^	88.84^a^	87.79^a^	80.58	1.04	<0.001
Glycine	79.56^c^	83.83^b^	90.67^a^	90.47^a^	89.16^ab^	77.90^c^	94.18^a^	82.40^c^	92.37^a^	91.67^a^	87.22	0.96	<0.001
Proline	74.11^de^	86.99^ab^	87.91^ab^	90.18^a^	74.20^de^	78.19^cd^	82.92^bc^	70.89^e^	79.60^cd^	86.28^ab^	81.13	1.05	<0.01
Serine	72.76^cd^	69.92^d^	76.70^bcd^	75.23^cd^	81.59^abc^	71.40^d^	90.05^a^	81.90^abc^	86.10^ab^	86.98^a^	79.26	1.26	<0.001
Tyrosine	88.86^ab^	86.11^abc^	85.79^abc^	84.58^bc^	89.01^ab^	81.48^c^	91.66^a^	80.68^c^	84.03^bc^	85.13^abc^	85.73	0.74	0.015

Basal endogenous losses (g/kg dry matter intake) averaged as CP, 17.91; Arg, 0.68; His, 0.24; Ile, 0.29; Leu, 0.57; Lys, 0.43; Met, 0.14; Phe, 0.46; Thr, 0.62; Trp, 0. 15; Val, 0.37; Ala, 0.52; Asp, 1.62; Cys, 0.15; Glu, 1.21; Gly, 1.34; Pro, 0.55; Ser, 0. 60; Tyr, 0.12. Detailed basal endogenous loss data are presented in Supplement 3.

a–e Means in the same row with no letter the same letter are not different at p<0.05.

SEM, standard error of the mean.

**Table 7:** Stepwise regression equations for SID of CP, Lys, Met, Thr and Trp based upon the chemical characteristics of the 10 rice bran samples (as-fed basis, %)

Items	Prediction equation	RSD	R^2^	p-value
SID_CP_	SID_CP_ = 135.252–29.780TP	4.16	0.60	0.008
SID_CP_	SID_CP_ = 974.032–29.805TP–9.946DM	2.44	0.88	<0.001
SID_Lys_	SID_Lys_ = 97.526–9.598TP	1.67	0.50	0.023
SID_Lys_	SID_Lys_ = 420.943–9.608TP–3.662DM	1.06	0.82	0.002
SID_Lys_	SID_Lys_ = 471.278–9.267TP–4.245DM+1.401DV	0.77	0.92	0.001
SID_Met_	SID_Met_ = 122.281–2.712CP	4.08	0.51	0.020
SID_Thr_	SID_Thr_ = 30.752+2.975GE	2.57	0.82	<0.001
SID_Thr_	SID_Thr_ = 51.864+2.204GE–1.324CF	1.21	0.97	<0.001
SID_Trp_	SID_Trp_ = 76.344–19.045Ca	2.34	0.78	<0.001
SID_Trp_	SID_Trp_ = 84.769–21.405Ca–0.357NDF	1.81	0.86	0.040
SID_Trp_	SID_Trp_ = 73.912–23.268Ca–0.336NDF+0.318TS	1.10	0.96	0.011

DVs take values of 0 and 1, which in this study correspond to defatted rice bran (0) and full-fat rice bran (1) respectively.

p<0.05 means significant difference; p<0.01 means extremely significant difference.

SID, standardized ileal digestibility; CP, crude protein; RSD, relative standard deviation; R^2^, R-square; TP, total phosphorus; DM, dry matter; DV, dummy variable; GE, gross energy; CF, crude fiber; Ca, Calcium; NDF, neutral detergent fiber; TS, total starch.

## SUPPLEMENTARY MATERIAL

**Supplement 1**. Stepwise regression equations for SID of CP, Lys, Met, Thr and Trp based upon the chemical characteristics of the 7 defatted rice bran samples (as fed basis, %).

**Supplement 2**. Correlation coefficients among chemical composition and the SID of the crude protein and first four limiting amino acids of the 7 defatted rice bran samples.

**Supplement 3**. Endogenous losses of crude protein and amino acids in growing pigs in the experiment (%).
