# Supplementary material for: Determination and prediction of amino acid digestibility in rice bran for growing pigs
Source: Anim Biosci. 2025 Aug 25;39(2):250280. doi: 10.5713/ab.25.0280 (PMC12877389; doi:10.5713/ab.25.0280)
Supplement: Supplementary file 1 [file ab-25-0280-Supplementary-1.pdf]

SUPPLEMENTARY MATERIAL

Supplement 1. Stepwise regression equations for SID of CP, Lys, Met, Thr and Trp based upon the chemical characteristics of the 7 defatted rice bran samples (as fed basis, %)

Items	Prediction equation	RSD	R^2	*p*-value
SID_{CP}	SID_{CP}=168.157-46.930TP	3.676	0.77	0.009
SID_{Lys}	SID_{Lys}=116.523-2.386CP	1.043	0.82	0.005
SID_{Met}	SID_{Met}=120.544-3.903Ash	3.037	0.69	0.012
SID_{Thr}	SID_{Thr}=149.165-4.721CP	1.826	0.86	0.003
SID_{Thr}	SID_{Thr}=148.403-3.816CP-1.309Ash	1.137	0.96	0.002
SID_{Trp}	SID_{Trp}=107.094-3.654Ash	2.339	0.78	0.003
SID_{Trp}	SID_{Trp}=102.656-3.616Ash+0.527ADF	1.814	0.86	0.001
SID_{Trp}	SID_{Trp}=119.156-1.912Ash+0.995ADF-19.952TP	1.102	0.96	<0.001

SID, standardized ileal digestibility; CP, crude protein; DM, dry matter; EE, ether extract; Lys, Lysine; Thr, Threonine; Trp, Tryptophan; R^2, R-square; RSD, relative standard deviation.

$p<0.05$ means significant difference; $p<0.01$ means extremely significant difference.
