# Supplementary material for: Determination and prediction of amino acid digestibility in rice bran for growing pigs
Source: Anim Biosci. 2025 Aug 25;39(2):250280. doi: 10.5713/ab.25.0280 (PMC12877389; doi:10.5713/ab.25.0280)
Supplement: Supplementary file 2 [file ab-25-0280-Supplementary-2.pdf]

Supplement 2. Correlation coefficients among chemical composition and the SID of the crude protein and first four limiting amino acids of the 7 defatted rice bran samples

	DM	GE	CP	EE	ASH	CF	NDF	ADF	Ca	TP	TS	SID_{CP}	SID_{Lys}	SID_{Met}	SID_{Thr}
GE	-0.913**														
CP	0.771*	-0.702													
EE	-0.928**	0.948**	-0.637												
ASH	0.484	-0.722	0.488	-0.602											
CF	0.311	-0.189	0.813*	-0.092	0.225										
NDF	0.482	-0.300	0.878**	-0.246	0.137	0.962**									
ADF	0.185	-0.063	0.657	0.060	-0.029	0.888**	0.888**								
Ca	0.317	-0.592	-0.019	-0.562	0.792*	-0.409	-0.450	-0.596							
TP	0.313	-0.415	0.662	-0.268	0.776*	0.725	0.592	0.519	0.259						
TS	0.526	-0.538	-0.062	-0.627	0.013	-0.595	-0.383	-0.503	0.424	-0.517					

SID_{CP}	-0.647	0.680	-0.721	0.555	-0.795*	-0.592	-0.560	-0.423	-0.383	-0.878**	0.130				
SID_{Lys}	-0.770*	0.751	-0.906**	0.725	-0.654	-0.689	-0.738	-0.537	-0.215	-0.765*	0.028	0.847*			
SID_{Met}	-0.751	0.861*	-0.763*	0.820*	-0.863*	-0.429	-0.450	-0.235	-0.565	-0.748	-0.129	0.841*	0.924**		
SID_{Thr}	-0.778*	0.758*	-.924**	0.698	-0.728	-0.718	-0.733	-0.460	-0.266	-0.810*	0.085	0.886**	0.942**	0.890**	
SID_{Trp}	-0.322	0.553	-0.195	0.494	-0.924**	0.049	0.156	0.358	-0.899**	-0.595	-0.059	0.623	0.408	0.683	0.522

*means significant difference ($p<0.05$); **means extremely significant difference ($p<0.01$).

SID_{CP}, SID_{Lys}, SID_{Met}, SID_{Thr}, and SID_{Trp}, SID of CP, Lys, Met, Thr and Trp, respectively.

Supplement 3. Endogenous losses of crude protein and amino acids in growing pigs in the experiment (%)

**Items**	**Nitrogen-free diet**						**Min**	**Max**	**Mean**	**SD**	**Z-score of Min**	**Z-score of Max**
	**1**	**2**	**3**	**4**	**5**	**6**						
CP, %	18.08	14.28	16.78	19.97	23.96	14.36	14.28	23.96	17.91	3.684	-0.983	1.643
Indispensable amino acids, %												
Arginine	0.82	0.68	0.76	0.72	0.57	0.52	0.52	0.82	0.68	0.115	-1.377	1.235
Histidine	0.23	0.26	0.23	0.23	0.23	0.25	0.23	0.26	0.24	0.012	-0.901	1.632
Isoleucine	0.19	0.29	0.27	0.29	0.33	0.39	0.19	0.39	0.29	0.066	-1.535	1.469
