# Supplementary material for: Determination and prediction of amino acid digestibility in rice bran for growing pigs
Source: Anim Biosci. 2025 Aug 25;39(2):250280. doi: 10.5713/ab.25.0280 (PMC12877389; doi:10.5713/ab.25.0280)
Supplement: Supplementary file 3 [file ab-25-0280-Supplementary-3.pdf]

SID_{CP}	-0.647	0.680	-0.721	0.555	-0.795*	-0.592	-0.560	-0.423	-0.383	-0.878**	0.130				
SID_{Lys}	-0.770*	0.751	-0.906**	0.725	-0.654	-0.689	-0.738	-0.537	-0.215	-0.765*	0.028	0.847*			
SID_{Met}	-0.751	0.861*	-0.763*	0.820*	-0.863*	-0.429	-0.450	-0.235	-0.565	-0.748	-0.129	0.841*	0.924**		
SID_{Thr}	-0.778*	0.758*	-.924**	0.698	-0.728	-0.718	-0.733	-0.460	-0.266	-0.810*	0.085	0.886**	0.942**	0.890**	
SID_{Trp}	-0.322	0.553	-0.195	0.494	-0.924**	0.049	0.156	0.358	-0.899**	-0.595	-0.059	0.623	0.408	0.683	0.522

*means significant difference ($p<0.05$); **means extremely significant difference ($p<0.01$).

SID_{CP}, SID_{Lys}, SID_{Met}, SID_{Thr}, and SID_{Trp}, SID of CP, Lys, Met, Thr and Trp, respectively.

Supplement 3. Endogenous losses of crude protein and amino acids in growing pigs in the experiment (%)

**Items**	**Nitrogen-free diet**						**Min**	**Max**	**Mean**	**SD**	**Z-score of Min**	**Z-score of Max**
	**1**	**2**	**3**	**4**	**5**	**6**						
CP, %	18.08	14.28	16.78	19.97	23.96	14.36	14.28	23.96	17.91	3.684	-0.983	1.643
Indispensable amino acids, %												
Arginine	0.82	0.68	0.76	0.72	0.57	0.52	0.52	0.82	0.68	0.115	-1.377	1.235
Histidine	0.23	0.26	0.23	0.23	0.23	0.25	0.23	0.26	0.24	0.012	-0.901	1.632
Isoleucine	0.19	0.29	0.27	0.29	0.33	0.39	0.19	0.39	0.29	0.066	-1.535	1.469

Leucine	0.60	0.56	0.52	0.35	0.68	0.73	0.35	0.73	0.57	0.134	-1.666	1.150
Lysine	0.61	0.37	0.28	0.46	0.51	0.34	0.28	0.61	0.43	0.122	-1.210	1.499
Methionine	0.16	0.14	0.07	0.14	0.22	0.09	0.07	0.22	0.14	0.055	-1.290	1.558
Phenylalanine	0.32	0.43	0.44	0.42	0.51	0.62	0.32	0.62	0.46	0.100	-1.389	1.600
Threonine	0.58	0.81	0.72	0.42	0.70	0.51	0.42	0.81	0.62	0.146	-1.419	1.271
Tryptophan	0.15	0.13	0.12	0.13	0.10	0.09	0.09	0.15	0.12	0.021	-1.403	1.255
Valine	0.44	0.52	0.34	0.40	0.33	0.19	0.19	0.52	0.37	0.113	-1.627	1.303
Dispensable amino acids, %												
Alanine	0.53	0.60	0.73	0.42	0.35	0.48	0.35	0.73	0.52	0.133	-1.260	1.562
Aspartate	1.52	1.75	1.57	1.70	1.54	1.64	1.52	1.75	1.62	0.092	-1.063	1.402
Cystine	0.24	0.09	0.15	0.19	0.09	0.14	0.09	0.24	0.15	0.060	-1.060	1.596
Glutamine	1.30	1.07	1.06	1.19	1.29	1.36	1.06	1.36	1.21	0.125	-1.202	1.145
Glycine	1.27	1.25	1.46	1.46	1.27	1.33	1.25	1.46	1.34	0.098	-0.875	1.248
Proline	0.79	0.56	0.48	0.42	0.39	0.64	0.39	0.79	0.55	0.150	-1.046	1.604
Serine	0.62	0.55	0.58	0.54	0.65	0.64	0.54	0.65	0.60	0.047	-1.177	1.185
Tyrosine	0.49	0.31	0.50	0.25	0.19	0.43	0.19	0.50	0.36	0.130	-1.298	1.042
